# Case Report: Application and Limitations of a Plant-Based Diet Formulated for a Cat With Feline Lower Urinary Tract Disease

**DOI:** 10.3389/fvets.2021.658265

**Published:** 2021-04-09

**Authors:** Sarah A. S. Dodd, Caitlin Grant, Sarah K. Abood, Adronie Verbrugghe

**Affiliations:** ^1^Department of Clinical Studies, Ontario Veterinary College, University of Guelph, Guelph, ON, Canada; ^2^Department of Population Medicine, Ontario Veterinary College, University of Guelph, Guelph, ON, Canada

**Keywords:** crystalluria, feline nutrition, struvite, urethral obstruction, vegan

## Abstract

A 2-year-old male castrated domestic shorthair cat was presented for recommendations for dietary management of chronic FLUTD using a strictly plant-based diet as per the stipulations of the cat's owner. The cat had a history of urethral obstruction of unknown etiology, persistent marked struvite crystalluria, and persistent inappropriate elimination. Commercial plant-based products meeting the nutritional recommendations for maintenance of adult cats with the lowest concentration of struvite precursors were identified, but the cat would not eat them. At the request of the client, a homemade plant-based diet was formulated with the intention of increasing water intake and promoting acidic, dilute urine. Urine concentration was able to be decreased somewhat and struvite crystalluria resolved, but the urine remained more alkaline than intended. The cat clinically improved and no further FLUTD episodes were reported by the client.

## Introduction

Feline lower urinary tract disorders (FLUTD) are common, potentially life-threatening problems in cats (1). Nearly 20% may be attributable to urethral obstruction, with 10–30% of obstructions attributable to urolithiasis (1–3). One of the most common uroliths are magnesium ammonium phosphate (struvite) (4, 5). Struvite has also been reported to be a common component of urethral plugs (6). Risk factors for FLUTD include breed, sex, reproductive status, age, body condition score (BCS), indoor and outdoor access, activity, number of pets in the household, potential stressors, litter management, diet, and water intake (1, 7). The focus of the case presented here is on management of nutritional risk factors.

## Case Presentation

A 2-year-old male castrated domestic shorthair cat was presented to his veterinarian for suspect urethral obstruction. On presentation, he was documented to have a BCS of 3 on a 5-point scale, weighing 4.85 kg. A full, hard bladder was palpated, and white, gritty material was noted at the tip of his penis. Pre-anesthetic bloodwork revealed few abnormalities (see Table 1). A catheter was passed with some resistance, allowing emptying of the bladder. Copious amounts of “sand” were present in the urine. Urine pH was 8.0, cocci bacteria and large quantities of struvite crystals were detected (Table 2). Due to the presence of struvite crystals in the urine and small uroliths visualized by ultrasound, a canned feline veterinary therapeutic diet formulated for dissolution and prevention of struvite uroliths was recommended (Table 3). Previously, the cat had been fed commercial plant-based diets (Table 3). The cat was discharged with amoxicillin-clavulanic acid (62.5 mg Q12h PO, 10 days), prazosin (0.5 mg Q12h PO, 15 days, then 0.5 mg Q24h, 15 days), buprenorphine (0.08 mg Q8h PO, 16 days, tapering to 0.04 mg Q24h as required), and robenacoxib (6 mg PO SID, 6 days). Alprazolam (0.5 mg Q12h, 14 days) was also prescribed for underlying anxiety. The cat re-presented twice over the following 5 weeks with acute difficulty urinating, vocalizing, straining, and urinating outside of the litter box. No abnormalities were detected on physical examinations and urinalyses revealed improvement in urine acidity with fewer struvite crystals. Against recommendations, the client discontinued the therapeutic food as it contained animal products, and the cat was returned to his previous commercial plant-based diet.

**Table 1:** Hematology and serum biochemistry at time of urethral obstruction.

**Parameter**	**Units**	**Result**
**Hematology**		
RBC	x10^12^/L	9.09
HCT	%	40.3
HGB	g/dL	11.8
MCV	fL	44.3
MCH	Pg	13.0
MCHC	g/dL	29.3
RDW	%	22.7
Retic	K/μL	8.2
WBC	x10^9^/L	14.61
Neu	x10^9^/L	11.01
Lym	x10^9^/L	2.91
Mono	x10^9^/L	0.31
Eos	x10^9^/L	0.14
Baso	x10^9^/L	0.24
PLT	K/ μL	397
MPV	fL	15.8
Plateletcrit	%	0.63
**Biochemistry**		
Glucose	mmol/L	19.26
Creatinine	mmol/L	122
Urea	mmol/L	7.0
Total protein	g/L	63
Albumin	g/L	32
Globulin	g/L	31
Albumin/globulin	Ratio	1.0
ALT	U/L	38
ALKP	U/L	38
Na	mmol/L	155
K	mmol/L	8.0
Na/K	Ratio	19
Cl	mmol/L	118
Osm	mmol/kg	330

**Table 2:** Selected urinalysis characteristics from time of urethral obstruction to end of follow-up.

**Parameter**	**Date**										
	**12/3/19**	**25/3/19**	**18/4/19**	**1/8/19**	**1/10/19**	**29/10/19**	**8/1/20**	**27/1/20**	**14/2/20**	**20/3/20**	**28/4/20**
Diet	Kibble B and C, Canned B	Therapeutic canned	Kibble B and C				Homemade diet		Kibble A		Kibble A, Formulated diet
Weight (kg)	4.85	NR	5.03	4.30			4.51			5.80	
BCS (/9)	5	NR	5	5			5			6	
Collection method	Catheterization	Free flow		Cystocentesis			Free flow				Cystocentesis
Urine specific gravity	1.040	1.032	1.018	1.030	1.065	1.025	1.060	1.060	1.060	1.040	1.038
pH	8.0	6.0	8.0	8.5	7.5	8.0	7.0	7.0	6.0	7.0	7.0
Protein	2+	2+	–	1+	1+	2+	1+	1+	1+	Trace	Trace
Blood	4+	–	–	3+	–	–	–	–	–	-	–
Crystals	10–16 S/hpf	Occasional S	10–20 S/hpf	1–5 S/hpf	3–5 S/hpf	Minor amorphous debris	10–20 S/hpf	>40 S/hpf	1–10 S/hpf	1–10 S/hpf	–
Red blood cells	4+	N	20–30/hpf	50–75/hpf	15–20/hpf	N	0–2/hpf	N	N	N	<1/hpf
White blood cells	1+	2–4/hpf	10–15	0–2/hpf	N	N	N	N	N	N	<1/hpf
Bacteria	1+ cocci	1+ cocci	Chains	N	N	N	N	1+ cocci	N	1+ cocci	N

–*, negative; S, struvite; NR, not reported*.

**Table 3:** Profile of specific nutrients of interest in commercial plant-based feline diets, compared to the recommended therapeutic diet.

	**Target**	**Recommended veterinary therapeutic diet**	**Initial diet**			**Recommended commercial diets**		**Homemade diets**	
			**Plant-based canned A**	**Plant-based kibble A**	**Plant-based kibble B**	**Plant-based canned C**	**Plant-based kibble C**	**Client's homemade diet**	**Formulated homemade diet**
Energy (kcal/100 g)	–	81.5	109.9	386.1	441.3	407.5	98.7	101.6	123.1
Base excess (mmol/kg DM)	<250	422	−51	−180	−160	−172	−72	−45	−398
**Nutrient (g/100 kcal)**									
Moisture	Moist	100.0	64.6	16.1	11.8	75.5	10.9	76.3	54.5
Protein	7.0–9.0	9.8	7.7	8.3	6.7	8.6	7.0	6.1	8.2
Methionine	0.10–0.38	0.18	0.11	0.18	0.16	0.13	0.17	0.12	0.32
Methionine + Cystine	>0.2	0.31	0.15	0.24	0.24	0.16	0.25	0.15	0.54
Taurine	>0.05	0.11	0.03	0.03	0.05	0.03	0.06	0.06	0.24
Fat	2.3–4.6	4.3	2.3	3.0	4.9	2.2	3.4	5.1	4.2
EPA + DHA	> 0.05	0.17	0.00	0.01	0.01	0.00	0.01	0.01	0.16
Calcium	0.14–0.30	0.26	0.30	0.20	0.28	0.16	0.18	0.24	0.28
Phosphorus	0.13–0.26	0.23	0.26	0.18	0.22	0.16	0.13	0.14	0.26
Magnesium	0.01–0.02	0.01	0.05	0.04	0.04	0.05	0.02	0.04	0.02
Sodium	0.07–0.14	0.33	0.12	0.04	0.08	0.08	0.17	0.04	0.18
Potassium	0.15	0.15	0.44	0.17	0.28	0.44	0.16	0.03	0.39
Chloride	>0.20	0.36	0.25	0.14	0.25	0.20	0.26	0.06^*^	0.22^*^
Sulfur	>0.05	NM	0.09	0.11	0.09	0.11	0.12	0.15^*^	0.15^*^

* *Likely underestimated as content is not reported in the USDA database or Canadian Nutrient File for most food products NM, not measured*.

One month later, the cat was referred to the Ontario Veterinary College Health Sciences Centre Clinical Nutrition Service for consultation regarding plant-based dietary management for FLUTD. The owner was vegan and required that they feed the cat a plant-based diet in order to comply with their ethics. On presentation, the cat had a BCS of 5/9 (8), weighing 5.0 kg. Nutritional evaluation revealed risk factors based on the WSAVA Nutritional Evaluation Checklist (9), including living in a multi-cat house, ongoing urinary signs, and being fed an unconventional diet. The cat's resting energy requirement (RER) was estimated using: (BW^0.75^*70 kcal) = 250 kcal (10). Given the cat's activity, current bodyweight and BCS, his daily energy intake was estimated to be RER*1.2 = 300 kcal. A recommendation was made for a commercial feline veterinary therapeutic diet formulated to manage signs of FLUTD (Table 3), which was declined by the client as it contained animal products. After discussing the challenges related to management with a plant-based diet, commercial plant-based diets were identified as alternative strategies. While detailed nutrient profiles are not typically available for many commercial plant-based diets available in Canada, nutrient analyses had been performed as part of a research project conducted by the authors (11). Diets meeting the AAFCO adult maintenance recommendations with the lowest concentration of struvite precursors were identified (Table 3). Based on energy density, food doses were recommended to combine kibble and canned food in a 50:50 ratio. The recommendation was made for the cat to be fed 35 g (143 kcal) kibble and 150 g (148 kcal) canned food, allowing for 10 kcal (3% of daily energy intake) to be given as treats or snacks. The client was advised on the energy and nutrient content of suitable treats and snacks. Recommendations were made to encourage water intake, feed small, frequent meals, and to supplement the diet with eicosapentaenoic acid and docosahexaenoic acid (EPA+DHA) at a dose equivalent to 0.5% of the diet on a dry matter basis (7). Recommendations to increase water intake included adding water to food, adding palatants to water, providing multiple water dishes in different locations, different materials (e.g., ceramic or metal), offering wide dishes to avoid whisker stimulation, and drinking fountains.

Three months later, the cat re-presented to his veterinarian for inappetence and weight loss. His bodyweight was recorded at 4.30 kg, though BCS was still documented as 3 on a 5-point scale. According to dietary anamnesis the cat would not eat the canned diet, nor accept soaked kibble or the DHA+EPA supplement recommended by the Clinical Nutrition Service, and he had continued to be fed the commercial plant-based diet he had been fed prior to the urethral obstruction (Table 3). Urine was collected by the client using non-absorbent litter (Table 2). A veterinary therapeutic dissolution diet was again discussed with the client as being the most evidence-based solution to mitigate risk of recurrent FLUTD signs, but the client declined this recommendation. Mirtazapine (2 mg Q24–48h as required) was prescribed to stimulate food intake. Though no dietary change was implemented, recheck examination 3 weeks later revealed a slight increase in body weight from 4.30 to 4.43 kg. Four months later, the cat re-presented for decreased urine output. At that time his body weight was 4.51 kg and his BCS was documented as 5 on a 9-point scale. Free-flow urine was examined again (Table 2), and a feline veterinary therapeutic dissolution diet was recommended, but again declined. The client elected to try to increase water intake and began preparing a home-cooked plant-based diet (Table 4).

**Table 4:** Homemade feline diet recipe, ingredients listed from highest to lowest inclusion (by weight).

**Client's homemade diet**	**Formulated diet**
Firm tofu	Pumpkin
Brown rice	Tofu
Red lentils	Carrots
Sweet potato	Soy protein isolate
Carrots	Chestnuts
Celery	Beets
Pumpkin	Beyond beef^®^
Green peas	Nutritional yeast
Green beans	Tomato paste
Vegetable oil	Balance IT^®^ Feline supplement
Apple cider vinegar	Sunflower oil
Olives	Pumpkin seeds
Kelp powder	Omega-3 fatty acid oil
Spirulina or chlorella	Olives
Nutritional yeast	Desiccated coconut
Dried parsley	Arachidonic acid supplement
Catnip	Basil
Flax seeds	
Vegecat^TM^ supplement	

Three months later, the cat was re-presented to the Clinical Nutrition service for management of inappetence. Despite the client's concerns about the cat's intake, on presentation the cat weighed 5.8 kg with a BCS of 6 on a 9-point scale, his heaviest recorded weight. Evaluation of the home-cooked plant-based diet using diet formulation software (Balance IT^®^) revealed the diet to be deficient in numerous nutrients, most notably meeting 0% of the cat's arachidonic acid requirement, while it provided iodine over 8 times the maximum recommended dose (Table 3). On a daily basis, the cat was fed: 1 tbsp homemade food, 2 tbsp of vegetables (pumpkin, green lentils, peas, squash, green beans, or corn), ¾ cup of kibble B, 1/8 tsp enzyme supplement, 250 mg methionine, and 1/8 tsp psyllium. The diet recipe was reported to have been acquired from a friend and it was unknown where the dosing had been acquired for the supplementation of enzymes, methionine, or psyllium. Treats included commercial plant-based deli slices, non-dairy cheese, potato chips, coconut yogurt, non-dairy sour cream, and dried nori seaweed. The client was made aware that the cat was slightly overweight and that his estimated daily caloric intake was more than sufficient to maintain his current bodyweight. Concern for inappetence or inadequate intake was not necessary. As the cat would not eat a canned food or soaked kibble, it was suggested to combine the kibble with preferred plant-based treats and foods the cat would eat, while also increasing water intake. The client was advised not to feed more than 10% of the daily caloric allotment from foods other than the kibble. Additionally, the methionine dose was increased from 250 to 500 mg to acidify the urine.

One month later, the cat was eating the recommended food, but struvite crystalluria persisted and the owner was concerned about inability to increase water intake on the dry extruded diet. The cat was re-presented to the Clinical Nutrition Service, weighing 5.8 kg with a BCS of 6 on a 9-point scale. A homemade plant-based diet was designed using web-based formulation software^1^ (Tables 3, 4) to meet the AAFCO nutrient profile for adult maintenance with low concentrations of struvite precursors, and utilized ingredients intended to promote urine acidity (12). Acidification of the urine was predicted by calculation of the base excess of the diet (13). Base excess in the food was calculated using the equation: Base excess (mmol/kg DM) = 2[Ca]+2[Mg]+[Na]+[K]−2[P]−2[Met]−[Cl] (13). Plant-based fatty acid supplementation was recommended, with plant-based arachidonic acid and EPA+DHA. As the cat was slightly overweight, a conservative energy intake was recommended to just meet his RER of 250 kcal to prevent further weight gain. The next urinalysis performed revealed a stable USG and pH, though struvite crystals were absent. The client was encouraged to continue regular rechecks with their family veterinarian, and feedback from the client 9 months later revealed the cat was doing well, he continued to eat a combination of the homemade food and commercial plant-based kibble, his urine was consistently free of struvite crystals, pH maintained between 6.5–7.0, and the USG below 1.040. The owner reported no further concerns with inappetence, no changes in bodyweight, and the cat had suffered no further FLUTD episodes.

## Discussion

The case report presented here was of a cat with chronic FLUTD of nearly 1-year duration. A complicating factor was the client's request for the diet to be plant-based. There was a lack of client adherence to veterinary therapeutic diet recommendations, resulting in multiple relapses. This case report proposes nutritional counseling and development of a nutrition support plan that fits both the patient's nutritional requirements as well as the owner's lifestyle, habits and beliefs.

Cats evolved consuming a diet comprised exclusively of prey, resulting in evolution of anatomical, physiological, and metabolic adaptations to utilize animal tissues (14, 15). These unique adaptations have resulted in the cat being classified as an obligate carnivore (16). With recent interest being shown in feeding plant-based diets to dogs and cats (17), there has been discussion as to the practical and ethical considerations of whether carnivorous animals require animal tissues, *per se*, or if their exceptional nutritional requirements can be met with animal-free diets enriched with key essential nutrients (18, 19). In addition to the challenges of meeting a cat's nutritional requirements from only plant, mineral, and synthetic ingredients, plant-based diets have also been proposed to be a potential risk factor for urinary alkalinization (20) and thus struvite urolithiasis, though this has yet to be substantiated.

In this case, the cat was first presented for urethral obstruction, though no underlying cause (urethral plug, urolith) was reported. The cat did not re-obstruct, but persistently demonstrated other signs of FLUTD. Signs of FLUTD include: dysuria, haematuria, pollakiuria, stranguria, inappropriate urination or obstruction (2, 3). There are multiple conditions that fall under the general term of FLUTD that may cause the aforementioned signs, including anatomic abnormalities, urolithiasis, urethral plugs, urethral obstruction, infection, neoplasia, idiopathic inflammation, obstruction, and reflex dyssynergia (21). Given that imaging revealed radiopaque debris in the bladder and urinalysis showed marked crystalluria, it was assumed that struvite, either as uroliths or as a component of a urethral plug, were the primary cause, though other causes of urethral obstruction were not ruled out.

The significance of struvite crystalluria in cats with FLUTD is not known, as healthy cats have been demonstrated to have asymptomatic struvite crystalluria (22, 23). However, a recent case report regarding a cat with feline idiopathic cystitis and marked struvite crystalluria (24) supported previous hypotheses that, in the absence of other aetiological agents, persistent urinary signs may be attributable to crystalluria (25–27).

Dietary factors, low water intake, and decreased urination frequency are considered the main risk factors for FLUTD (3, 5, 28). Thus, promoting large volumes of dilute urine, and simultaneously encouraging frequent urination are the key approaches for nutritional management. Addition of anti-inflammatory nutrients such as DHA and EPA may also be beneficial (7). In cases with urolithiasis, additional nutritional interventions are indicated. Considering that struvite crystals are composed of magnesium, ammonium and phosphate, high dietary levels of these nutrients are considered to be causative (1, 29). Additionally, other minerals that may affect urinary excretion of phosphorus, such as calcium and chloride may also contribute toward struvite crystalluria. For uroliths to form, the urine environment must favor precipitation of crystals and aggregation of crystals into stones. This requires a high concentration of solutes, urine pH conducive to precipitation of phosphorus and magnesium, presence of ammonia, and a relatively low urine volume (3). Thus, intervention for dissolution and prevention of struvite urolithiasis is targeted at reducing the formation of struvite crystals and/or impairing the ability of crystals to aggregate into stones (3).

Firstly, controlling intake, and thus, to a degree, excretion of precursor minerals can reduce the substrate for crystal formation (30). The contribution of urinary protein to struvite crystalluria has been less commonly reported but demonstrated *in vitro*, making consideration of dietary protein levels warranted to reduce protein excretion (31). Protein restriction is not required, but avoidance of protein in gross excess of the recommended intake may be prudent. Concentration of precursors is a component of struvite crystalluria. The presence of ammonia and a pH > 6.0 are required for precipitation to occur, as high urinary pH reduces the solubility of phosphorus and magnesium in the urine, allowing the ammonia to bind the minerals leading to precipitation as struvite crystals (29, 31). Manipulation of the urinary pH to maintain a slightly acidic (pH < 6.5) environment thus increases solubility of struvite, reducing crystal precipitation (30, 32). Urine pH can be manipulated by inclusion of acidic sulfur-containing amino acids and minerals such as phosphorus, chloride and sulfur, and restriction of basic minerals, such as calcium, magnesium, sodium, and potassium. Water is also a key consideration in cases with crystalluria and urolithiasis. In order to mitigate aggregation of crystals into stones, urine volume can be increased to reduce urine concentration and saturation and increase frequency of voiding (3, 33). By increasing voiding frequency, urine spends less time within the bladder, reducing the time during which uroliths can form and grow (3). High moisture diets are considered a key strategy in prevention of urolithiasis, regardless of composition (34). Manipulation of dietary sodium can also be used to increase voluntary water intake (35).

In this case, veterinary therapeutic diets were unacceptable to the client, due to the presence of animal products. Initially, commercial plant-based products with low magnesium content and base excess supportive of acidic urine formation were identified as alternative recommendations (36). First, canned products were suggested as an effective method of increasing water intake in cats (3, 37). Moisture content >73% has been reported to increase total water intake and urine volume while decreasing urine specific gravity (38). The canned food had similar magnesium content, but greater protein, sulfur amino acid and moisture content, and a lower base excess than the canned food previously fed (Table 3). It was hypothesized this would lead to more acidic and less concentrated urine. The cat found this canned diet unpalatable and would not eat it, so next an extruded product higher in salt (sodium chloride) was identified to increase water intake (39, 40). Additionally, it was recommended to feed multiple smaller meals to encourage more frequent drinking and reduce the magnitude of the post-prandial alkaline tide (41, 42). Finally, plant-based EPA+DHA and arachidonic acid supplements were recommended. Anti-inflammatory effect have been demonstrated for EPA and DHA, while arachidonic acid may help maintain water balance in the kidney, aside from being an essential nutrient for cats (43).

Palatability of the plant-based diets was an issue; the cat consistently refused to consume adequate amounts of diets identified as low risk for FLUTD, but preferred diets unconducive to acidification or production of dilute urine. As a result, the client elected to feed a home-prepared diet. Though the cat preferred to eat the client's home-prepared diet, it was unsuitable not only for dietary management of FLUTD but was also inadequate for adult maintenance as a result of numerous nutrient imbalances (Table 3). A new home-prepared diet was formulated with a nutrient profile more conducive for mitigation of FLUTD. As with the commercial diets, appropriate dietary protein, sulfur amino acids, EPA+DHA, and salt, and lower magnesium were prioritized. Reduction of urine pH was anticipated by manipulating the base excess of the diet and including sulfur-rich acidifying ingredients (12), while increased urine production was expected by increasing salt and recommendations to add water to the food and stimulate voluntary water intake. Initially, the cat's acceptance of the homemade diet formulated by the nutrition service was also limited, and the owner reported inappetence or disinterest in eating the diet. Over a 2 week period, minor modifications, such as inclusion of olives, tomato paste, and basil per the client's experience, improved the palatability of the diet and eventually the cat accepted the diet and maintained an adequate intake. No further episodes of FLUTD were reported and the client was pleased with the outcome.

None of the commercial PBD were specifically formulated for dissolution or prevention of struvite urolithiasis, nor had any undergone feeding trials to demonstrate either efficacy in promoting urinary tract health or suitability for feline adult maintenance.

Most veterinary therapeutic urinary diets undergo relative supersaturation testing to determine efficacy of dissolution or prevention of struvite uroliths (38, 44), but this testing has not been performed for the commercial plant-based or homemade diets. Instead, plant-based diets were selected for nutrient profiles as close as possible to one of several available therapeutic diets. Though the homemade diet was formulated specifically for struvite prevention, no guarantee of efficacy could be made. With the exception of a single timepoint, urinary pH was consistently neutral (7), not acidic (6–6.5) as intended. The urine concentration fluctuated greatly, but began to trend toward dilution once the homemade diet was initiated. This was not surprising, however, since the homemade diet was fed in conjunction with kibble and assorted treats, potentially contributing toward urinary alkalization. Furthermore, timing of urine collection was not recorded, nor whether urine samples were fasted or fed, all factors that influence urine pH. Though failure to acidify the urine was a clear limitation in the application of the homemade diet, resolution of clinical signs and improvement in other urinary parameters suggests adequate dietary modification to prevent FLUTD in this cat, at least during the 9 months of follow-up.

Ideally, any cat with a history of recurrent FLUTD and crystalluria should be monitored closely with repeat urinalyses every 3–6 months. Specifically, changes in pH (particularly increases in pH), increase in urine specific gravity, presence of protein in the urine or increases in crystalluria would warrant investigation and potentially a change to the diet. For a cat fed an unconventional diet, including a plant-based diet, further monitoring at 6-monthly intervals would be desired to ensure the cat is able to maintain general wellness by routine physical examination, complete blood count, and serum biochemistry.

## Conclusion

Management of FLUTD is complex and multifactorial, with appropriate nutrition and feeding a critical component. This case presented a greater challenge due to the client's preference for a plant-based diet, as there are no diets formulated and tested for management of FLUTD that do not contain animal ingredients. Commercial plant-based diets with nutrient profiles closest to that recommended for FLUTD were trialed but not accepted, culminating in formulation of a homemade plant-based diet. Unlike veterinary therapeutic diets that have undergone RSS testing and/or clinical trials, there is no evidence to support the efficacy of homemade formulations for mitigation of FLUTD, crystalluria, or urolithiasis. Nevertheless, 9 months post-intervention, the cat was reported by the client to be healthy and well.

## Data Availability Statement

The original contributions presented in the study are included in the article/supplementary material, further inquiries can be directed to the corresponding author/s.

## Ethics Statement

Ethical review and approval was not required for the animal study because this case report describes clinical treatment of a privately owned animal. Written informed consent was obtained from the owners for the participation of their animals in this study.

## Author Contributions

SD was veterinarian directly managing this case with CG's support. AV and SA supervised and mentored SD. SD wrote the report with assistance and feedback from CG, AV, and SA. All authors contributed to the article and approved the submitted version.

## Conflict of Interest

The authors declare that SD is the owner of Dodd Veterinary Services and provides both veterinary care and nutritional consultation to private clients and industry partners. CG holds the Nestlé Purina Professorship in Companion Animal Nutrition at the Ontario Veterinary College, is the owner of Grant Veterinary Nutrition Services and consults with Simmons Pet Food. SA is the owner of Sit, Stay Speak Nutrition LLC and provides nutrition consultation to industry partners. AV is the Royal Canin Veterinary Diets Endowed Chair in Canine and Feline Clinical Nutrition at the Ontario Veterinary College, serves on the Health and Nutrition Advisory Board for Vetdiet and has received honoraria and research funding from various pet food manufacturers and ingredient suppliers.

## Footnotes

^1^Balance IT^®^.
